# Maternal high-fat diet alters expression of pathways of growth, blood supply and arachidonic acid in rat placenta

**DOI:** 10.1017/jns.2013.36

**Published:** 2014-01-02

**Authors:** Marloes Dekker Nitert, Kanchan Vaswani, Melissa Hum, Hsiu-Wen Chan, Ryan Wood-Bradley, Sarah Henry, James A. Armitage, Murray D. Mitchell, Gregory E. Rice

**Affiliations:** 1 University of Queensland Centre for Clinical Research, Royal Brisbane and Women's Hospital Campus, Herston, QLD, Australia4029; 2 Department of Anatomy and Developmental Biology, Monash University, Clayton, VIC, Australia3800; 3 School of Medicine (Optometry), Faculty of Health, Deakin University, Waurn Ponds, VIC, Australia

**Keywords:** Placenta, High-fat diet, Arachidonic acid, LIM domain kinase 1, COX2, cyclo-oxidase 2, HFD, high-fat diet, LIMK1, LIM domain kinase 1, mRNA, messenger RNA

## Abstract

The high fat content in Western diets probably affects placental function during pregnancy with potential consequences for the offspring in the short and long term. The aim of the present study was to compare genome-wide placental gene expression between rat dams fed a high-fat diet (HFD) and those fed a control diet for 3 weeks before conception and during gestation. Gene expression was measured by microarray and pathway analysis was performed. Gene expression differences were replicated by real-time PCR and protein expression was assessed by Western blot analysis. Placental and fetal weights at E17.25 were not altered by exposure to the maternal HFD. Gene pathways targeting placental growth, blood supply and chemokine signalling were up-regulated in the placentae of dams fed the HFD. The up-regulation in messenger RNA expression for five genes *Ptgs2* (fatty acid cyclo-oxidase 2; COX2), *Limk1* (LIM domain kinase 1), *Pla2g2a* (phospholipase A2), *Itga1* (integrin α-1) and *Serpine1* was confirmed by real-time PCR. Placental protein expression for COX2 and LIMK was also increased in HFD-fed dams. In conclusion, maternal HFD feeding alters placental gene expression patterns of placental growth and blood supply and specifically increases the expression of genes involved in arachidonic acid and PG metabolism. These changes indicate a placental response to the altered maternal metabolic environment.

The highly vascularised placenta supplies nutrients to the developing fetus and epidemiological studies have shown that impaired fetal growth and the development of adult-onset disease are strongly associated with compromised placental structure and function. Converging lines of evidence now suggest that the placenta plays a significant role in intra-uterine programming by conveying critical growth-regulating signals between mother and fetus. Maternal overnutrition, which is increasingly common in the developed world, has been reported to alter placental function and structure and thereby the fetal growth trajectory^(^^1^^)^. In C57BL/6 mice, a high-fat diet (HFD) has been found to induce placental oxidative stress, causing vascular dysfunction and thereby disrupted nutrient transport^(^^1^^,^^2^^)^. Similar findings of altered vascular development have been reported in the placentae of Sprague–Dawley rats fed a HFD^(^^3^^)^. In Japanese macaques maintained on a chronic HFD, placental expression of a range of cytokines and Toll-like receptor 4 (TLR-4) is increased when compared with animals maintained on a normal diet^(^^4^^)^. Gene expression of specific targets in the placenta has been studied previously. Recently, it was reported that messenger RNA (mRNA) expression of GLUT1 (*Slc2a1*) is down-regulated whereas that of GLUT3 (*Slc2a3*) is up-regulated in placentae from HFD-fed rats^(^^5^^)^. Similarly, up-regulation of mRNA expression of the cationic amino acid transporter *Slc7a1* and down-regulation of the Na-dependent amino acid transporter *Slc38a4* is observed in the placentae from HFD-fed rats^(^^5^^)^. The mechanisms underlying the changes in placental morphology and gene expression are incompletely described. It is known, however, that HFD feeding increases the expression of imprinted genes such as the *Igf2r* gene^(^^6^^)^. This indicates decreased levels of methylation which may be secondary to the reported decreased expression levels of the DNA methyltransferases *Dnmt1* and *Dnmt3a*^(^^5^^)^.

In mice, a HFD exerts distinct effects on genome-wide placental gene expression^(^^6^^–^^8^^)^. Mao *et al.* reported that both a HFD and a low-fat diet have pronounced and specific effects on placental gene expression that are different for male and female fetuses, with larger changes observed in females^(^^7^^)^. Sexual dimorphic patterns were similarly observed in the expression and DNA methylation levels of imprinted genes in the placenta of another mouse model on a HFD^(^^6^^)^. When genome-wide gene expression was studied in this last model, the HFD altered the placental gene expression of both female and male fetuses but only a fraction of the genes overlapped between the sexes. While there have been reports on the effects of HFD feeding on mRNA expression of specific placental genes, there are no studies on the effects of maternal HFD feeding on global placental gene expression in the rat. The aim of the present study, therefore, was to characterise genome-wide placental gene expression to identify genes and pathways commonly affected by HFD feeding in male and female rat fetuses.

## Materials and methods

### Animals

Female Sprague–Dawley rats, aged 8–9 weeks, were obtained and allowed to acclimatise for 1 week before diet onset. The animals were maintained in a light-controlled environment (12 h light–12 h dark cycle; 24°C) throughout the study. After 1 week, female rats were randomly allocated to a hyperenergetic HFD (SF08-023; Specialty Feeds) or a control diet (SF09-091) (Table 1). The fat component of the HFD consisted of pork lard and rapeseed oil; in the control diet the fat component was rapeseed oil only. Both diets contained sucrose, wheat starch and dextrinised starch as sources of carbohydrates, although to different extents. The diets had similar contents of vitamins and minerals. After 3 weeks, the female rats were time-mated for 3 h with male Sprague–Dawley rats fed a control diet. This day was designated as embryonic day zero (E0). After mating, the dams were individually housed and maintained on their respective diets, having food and water *ad libitum* until killing at E17.25, a stage in pregnancy in which there is rapid fetal growth. Placentae were obtained and weighed, snap-frozen in liquid N_2_ and stored at –80°C. Approval was obtained from the School of Biomedical Sciences Animal Ethics Committee at Monash University (SOBSA/2008/39).

**Table 1.** Diet composition

	Control diet	High-fat diet
Protein (%)	19·4	23·3
Total fat (%)	7·0	23·5
Carbohydrates (%)	73·6	53·2
Digestible energy (MJ/kg)	16·1	19·1
Energy from protein (%)	21·0	21·0
Energy from lipids (%)	16·0	42·2
Energy from carbohydrates (%)	63·0	36·8
Fatty acid composition		
16 : 0 (%)	0·40	5·60
18 : 0 (%)	0·10	0·10
18 : 1 (%)	4·20	8·60
18 : 2 (%)	1·30	3·60
18 : 3 (%)	0·98	0·50
Total *n*-3 (%)	0·98	0·73
Total *n*-6 (%)	1·51	3·63
Total MUFA (%)	3·98	9·22
Total PUFA (%)	2·50	4·47
Total SFA (%)	0·50	9·83

### Gene expression microarray

A quantity of 30 mg placental tissue (wet weight) from one placenta per dam on the HFD (*n* 4) or the control diet (*n* 6) was homogenised with a mortar and pestle in liquid N_2_. RNA was isolated with the AllPrep DNA/RNA mini kit (Qiagen) according to the manufacturer's specifications. Total RNA was quantified and its quality assessed on a Bioanalyser (Agilent 2100). RNA samples with RNA integrity number >7, 260:280 ratio >2 and 260:230 ratio >1 were selected for microarray analysis.

Total RNA (500 ng) was converted and biotinylated with the Illumina TotalPrep RNA amplification kit (Illumina). Biotinylated samples were hybridised to the RatRef-12 Expression BeadChip (Illumina), incorporating twelve samples per chip. The chip was scanned on a BeadStation 500 System using Beadscan software v3.5.31. Gene expression data were normalised by probe-intensity transformation and normalisation (Lumi package for the analysis of Illumina microarray data in the bioconductor software suite^(^^9^^)^). Differences in expression between the HFD and control diets were calculated with the unpaired Student's *t* test, with *P* < 0·05 considered to be statistically significant. Pathway analysis on up- or down-regulated genes with a *P* < 0·05 was based on GO/KEGG by the Web-based Gene Set Analysis Toolkit (Webgestalt; http://bioinfo.vanderbilt.edu/webgestalt). A further pathway analysis was undertaken on a list of genes with a fold-change of 1·4 for up-regulated genes. Both pathway analyses were repeated with the DAVID algorithm (http://david.abcc.ncifcrf.gov) to ensure the validity of the pathway analyses.

### Quantitative real-time PCR

Validation of the microarray results for six genes with high fold-changes was performed by real-time PCR. RNA isolated from ten individual HFD placentae and ten individual control placentae (one placenta per dam) that included all the samples analysed by microarray was reverse transcribed into complementary DNA (QuantiTect cDNA synthesis kit; Qiagen). A total of six genes were selected for validation: five genes that were up-regulated (*Ptgs2*, *Pla2g2a*, *Serpine1*, *Itga1* and *Limk1*; encoding for cyclo-oxidase 2, phospholipase A2, Serpin1, integrin α-1 and LIM domain kinase 1, respectively) and one gene that was down-regulated (*Umps*; encoding for UMP synthetase). The up-regulated genes had a fold-change of >1·4 and *P* < 0·05. The down-regulated gene had a fold-change of <0·71 and *P* < 0·05. SYBR green assays were designed on the National Center for Biotechnology Information (NCBI) primer design tool. All assays spanned exon–exon junctions and primer specificity was verified by PrimerBlast (NCBI). Primer sequences can be found in Supplementary Table 1. Primer efficiency was established and dissociation curve analysis confirmed the presence of one PCR product at the expected melting temperature. Gene expression for β-actin (*Actb*) was used as the endogenous control. Real-time PCR was performed in a 20 µl volume with 10 µl SYBR green PCR master mix (Life Technologies), 20 ng complementary DNA and 300 nm forward and reverse primers, respectively in the StepOnePlus real-time PCR system (Life Technologies). Gene expression was compared with the comparative cycle threshold (C_T_) method (2^−ΔΔC^_T_).

### Western blot

Placental tissue (50 mg) was homogenised with a mortar and pestle, and lysed in ProteoJET Mammalian Cell Lysis Reagent with protease inhibitors (PhosSTOP Phosphatase Inhibitor Cocktail; Roche Applied Science). Protein concentration was determined by the bicinchoninic acid method with bovine serum albumin as the protein standard (Pierce, ThermoFisher Scientific). A quantity of 30 µg of total protein was loaded onto NuPAGE Bis-Tris Gel (4–12 or 12 %) (Life Technologies). Proteins were transferred onto PVDF (polyvinylidene fluoride) membranes (Millipore). The membrane was blocked in 5 % skimmed milk in PBS–Tween for 1 h at room temperature before overnight incubation with primary antibody for fatty acid cyclo-oxidase 2 (COX2) (sc-7951; Santa Cruz) and β-actin (A5316, SigmaAldrich) at 4°C. Secondary horseradish peroxidase (HRP)-conjugated antibodies (donkey anti rabbit HRP, Sigma 90545 and donkey anti mouse HRP, Santa Cruz Biotechnology 2314) were incubated for 1 h at room temperature in a Super Signal West Dura, extended duration (Pierce 34075) and the blots were developed for 5 min before scanning on the GS0800 imaging system (BioRad). For LIMK1, rabbit primary antibody (ab81046; Abcam) Secondary LI-COR^®^ antibody (goat anti-rabbit 800CW LI-COR^®^ 926-32211 and donkey anti-mouse 680LT LI-COR^®^ 926-68022) was incubated for 1 h at room temperature and protein was detected by the Odyssey Infrared Imaging System (LI-COR Biosciences). Protein expression was analysed by densitometry, with β-actin used to normalise for differences in protein loading.

### Statistical analysis

Individual placentae were investigated for all experiments, and each placenta was obtained from an independent litter. For all analyses, between-group comparisons of the means were made with unpaired Student's *t* test after confirming that the data were normally distributed. The microarray analyses were performed on six placentae from dams on the control diet and four placentae from dams on the HFD. The quantitative PCR replication experiments were carried out on RNA isolated from ten placentae per group. The Western blot analyses were performed on protein from ten placentae each for COX2, and on protein from nine placentae from the control diet group and six HFD placentae for LIMK1. Data were presented as mean values with their standard errors; *P* < 0·05 was considered to be statistically significant.

## Results

### Fetal and placental weights

Fetal and placental weights were similar in the control and HFD groups at E17.25.

Fetal weights were similar in rats on the control diet (0·69 (sem 0·02) g) and the HFD (0·68 (sem 0·03) g; *P* = 0·87) as were placental weights (control diet 0·36 (sem 0·02) *v.* HFD 0·35 (sem 0·02) g; *P* = 0·91). Maternal weights were not different at E17.25 between the diet groups (control diet 328·5 (sem 33·4) *v.* HFD 347·5 (sem 33·3) g; *P* = 0·69).

### Genome-wide gene expression

Microarray analysis of placental RNA from six dams on the control diet and four dams on the HFD yielded 825 differentially expressed genes with a cut-off of *P* < 0·05; none of the genes were differentially expressed after correction for multiple testing. Of the 825 genes, there were 457 annotated genes with 327 up-regulated and 130 down-regulated genes in the placentae of dams on the HFD (Supplementary Table 2). Pathway analyses were performed separately for up-regulated and down-regulated genes on the basis of the KEGG/GO database with two different algorithms, with the top ten pathways for each presented in Table 2.

**Table 2.** Pathway analysis of all up-regulated and down-regulated genes with *P* < 0·05 with the Webgestalt pathway analysis tool

Pathway (total number of genes in pathway)	O	E	R	*P*	Adjusted *P*
Up-regulated genes					
Metabolic pathways (1169)*	28	8·09	3·46	1·6 × 10^−8^	1·34 × 10^−6^
MAPK signalling (269)*	10	1·76	5·37	2·07 × 10^−5^	0·0009
1C pool by folate (18)*	3	0·12	24·10	0·0002	0·0028
Lysosome (124)*	6	0·86	7·00	0·0002	0·0028
Antigen processing and presentation (91)*	5	0·63	7·94	0·0004	0·0048
Alzheimer's disease (212)	8	1·47	5·46	0·0001	0·0021
Apoptosis (93)*	4	0·64	6·22	0·0040	0·026
Glycosphingolipid biosynthesis – ganglio series (15)*	2	0·10	19·28	0·0047	0·026
Chemokine signalling (178)	5	1·23	4·06	0·0082	0·035
Arachidonic acid metabolism (74)	3	0·51	5·86	0·0148	0·042
Down-regulated genes					
Alzheimer's disease (212)*	5	0·56	8·99	0·0003	0·0075
RNA degradation (77)*	3	0·20	14·85	0·0011	0·0081
Peroxisome (80)*	3	0·21	14·29	0·0012	0·0081
Proximal tubule bicarbonate reclamation (20)	2	0·05	38·11	0·0013	0·0081
Metabolic pathways (1169)*	9	3·07	2·93	0·0038	0·016
Leucocyte transendothelial migration (114)	3	0·30	10·03	0·0034	0·016
Tight junction (131)*	3	0·34	8·73	0·0061	0·018
Notch signalling (50)	2	0·13	15·24	0·0077	0·024
Basal cell carcinoma (53)	2	0·14	14·38	0·0086	0·024
Pathways in cancer (319)	4	0·84	4·78	0·0101	0·025

O, Observed number of genes; E, expected number of genes; R, O:E ratio; MAPK, mitogen-activated protein kinase.

* Pathways also found with the DAVID pathway analysis algorithm.

Pathway analysis was also performed on nineteen up-regulated genes with a fold-change larger than 1·4 in HFD-fed dams (Supplementary Table 3). The pathway analysis was based on the gene ontology database and seven pathways were found with the Webgestalt tool (Table 3); all of these were repeated with the DAVID pathway analysis tool. The main pathways included regulation of apoptosis, growth regulation, circulation and chemotaxis.

**Table 3.** Pathway analysis based on gene ontology in the Webgestalt pathway analysis tool with eighteen up-regulated genes with a fold-change >1·4 or down-regulated with a fold-change <0·7

Pathway (total number of genes in pathway)	O	E	R	*P*	Adjusted *P*
Regulation of apoptosis (729)	5	0·97	5·15	0·0021	0·044
Includes *Ptgs2* and *Itga1*					
Regulation of angiogenesis (59)	2	0·08	25·47	0·0027	0·044
Includes *Serpine1*					
Cell chemotaxis (47)	2	0·06	31·97	0·0017	0·044
Includes *Itga1*					
Regulation of anatomical structure size (347)	4	0·46	8·66	0·001	0·044
Includes *Ptgs2*, *Limk1* and *Itga1*					
Developmental growth (173)	3	0·23	13·03	0·0015	0·044
Includes *Ptgs2*, *Limk1* and *Serpine2*					
Blood circulation (204)	3	0·27	5·09	0·0023	0·044
Includes *Ptgs2* and *Itga1*					
Oxidoreductase activity, acting on single donors with incorporation of molecular oxygen (38)	2	0·04	47·65	0·0008	0·034
Includes *Ptgs2*					

O, Observed number of genes; E, expected number of genes; R, O:E ratio.

**Fig. 1.**
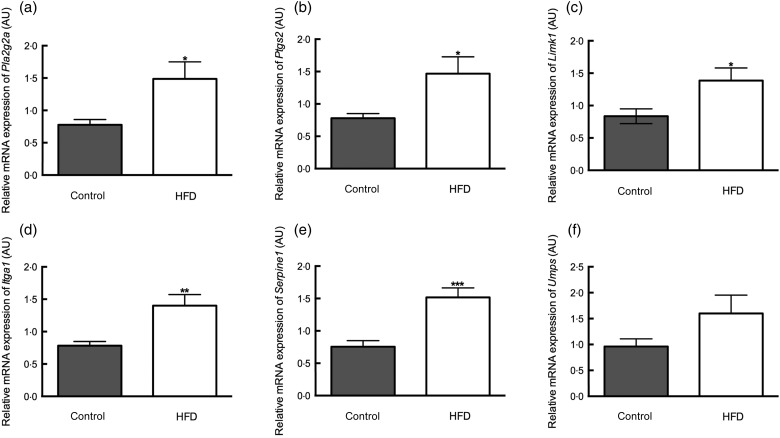
Replication of microarray results with real-time PCR for *Pla2g2a*, encoding for phospholipase A2 (a), *Ptgs2*, encoding for cyclo-oxidase 2 (b), *Limk1*, encoding for LIM domain kinase 1 (c), *Itga1*, encoding for integrin α-1 (d), *Serpine1*, encoding for Serpin1 (e) and *Umps*, encoding for UMP synthetase (f). mRNA, messenger RNA; AU, arbitrary units; HFD, high-fat diet. Values are means (*n* 10 placentae (one placenta per dam) in each group), with standard errors represented by vertical bars. Mean value was significantly different from that of the control group: *P < 0·05, **P < 0·01, ***P < 0·001.

The microarray results were confirmed by real-time PCR for five up-regulated genes and one down-regulated gene with high fold-changes: *Limk1*, *Itga1*, *Pla2g2a*, *Ptgs2*, *Serpine1* and *Umps*. These genes were selected based on their known functions and their prominence in the pathway analyses. Differential expression was observed for the genes that were up-regulated in placentae from HFD-fed dams, for example, *Limk1* (fold-change 4·1), *Itga1* (fold-change 1·8), *Pla2g2a* (fold-change 1·9), *Ptgs2* (fold-change 1·9), *Serpine1* (fold-change 2·0) but not for the down-regulated gene *Umps* (fold-change 1·7) (Fig. 1). We investigated if the change in mRNA expression translated to a change in protein expression for fatty acid COX2 (*EC* 1·44·99·1), which is encoded by *Ptgs2*, and the serine/threonine kinase LIMK1, which is encoded by *Limk1*.

### Protein expression

**Fig. 2.**
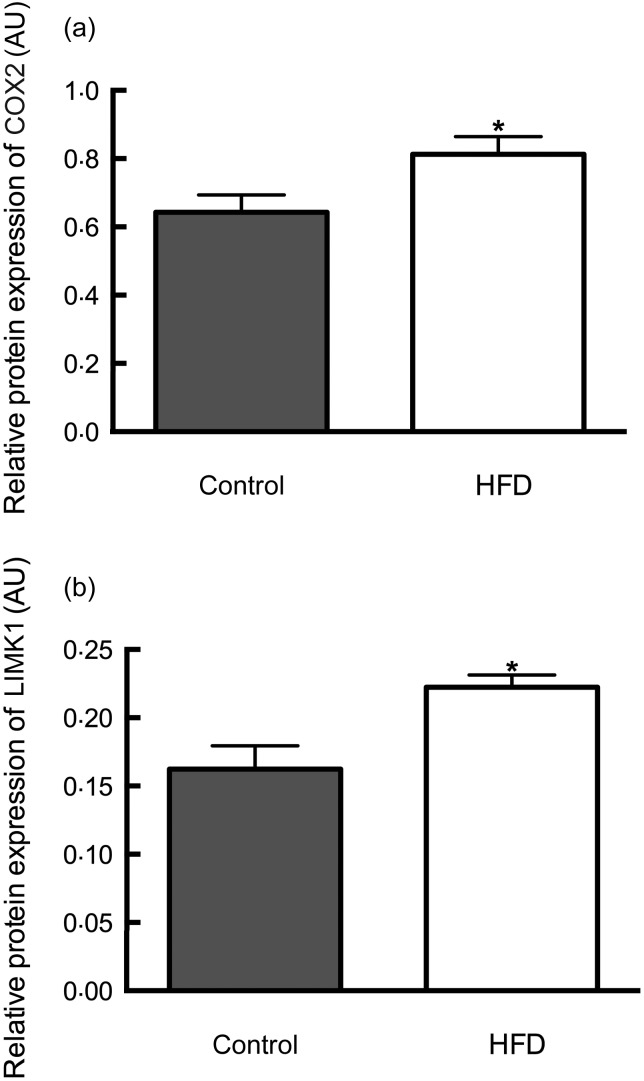
Placental protein expression of cyclo-oxidase 2 (COX2) (a) and LIM domain kinase 1 (LIMK1) (b). AU, arbitrary units; HFD, high-fat diet. Values are means (*n* 10 placentae (one placenta per dam) in each group for COX2; *n* 9 in control group and *n* 6 in HFD group for LIMK1), with standard errors represented by vertical bars. *Mean value was significantly different from that of the control group (*P* < 0·05).

Compared with controls, placentae from dams fed the HFD showed a 1·3-fold increase in protein expression of COX2 (control diet 0·64 (sem 0·050) *v.* HFD 0·81 (sem 0·051); *P* < 0·05) in line with the increased mRNA expression (Fig. 2(a)). Similarly, LIMK1 was found to have increased protein expression by 1·4-fold in placentae from HFD-fed dams (control diet 0·16 (sem 0·017) *v.* HFD 0·22 (sem 0·009); *P* < 0·05) (Fig. 2(b)).

## Discussion

The aim of the present study was to establish the effects of a maternal HFD on rat placental gene expression using a genome-wide approach. The HFD was associated with the differential expression of more than 800 placental probes, corresponding to 457 known genes. Many of the up-regulated genes in the HFD group were associated with signal transduction pathways, including those involving phospholipid-derived mediators, cytokines and chemokines. This is not unexpected given the known associations of HFD feeding with low-grade inflammation^(^^10^^,^^11^^)^. Metabolic pathways in the placenta were also significantly affected by the HFD. Regulation of metabolic pathways in the placenta by overnutrition and undernutrition has been reported previously in mouse models^(^^7^^,^^12^^)^, specifically, placental expression differences between male and female offspring of dams on a control diet for amino acid and carbohydrate metabolism pathways and for lipid metabolism in HFD-fed dams but not in metabolism pathways between the diets^(^^8^^)^. In addition, pathway analyses indicated that methylation may be altered by HFD feeding since mRNA expression of genes in the 1C pool by folate pathway was significantly altered. This is in accordance with the results obtained by Lin *et al.*^(^^5^^)^ who reported that expression levels of the DNA methyltransferases *Dnmt1* and *Dnmt3a* were decreased in the placenta of rats fed a HFD. In female offspring of mouse dams fed a HFD, decreased expression of *Dnmt3l* has been reported^(^^8^^)^. Decreased levels of methylation could result in increased levels of gene expression. In the present study, the HFD resulted in increased gene expression of more genes than the number of genes with decreased levels of gene expression.

The pathway analysis conducted including only the nineteen up-regulated genes with a fold-change of >1·4 indicated alterations in pathways that modulate angiogenesis and circulating blood as well as growth and development. Heightened expression of genes in these pathways could lead to increased growth of the placenta and thereby the fetus. HFD feeding in mice has been reported to increase offspring neonatal fat mass^(^^13^^)^. At E17.25 there were no differences in either placental or fetal weight, and although fetal weight increased by approximately 2·3 g between E17.25 and birth there were no effects on fetal weight caused by the maternal HFD (data not shown).

The pathway analysis of down-regulated genes yielded three pathways common to both pathway analyses: Alzheimer's disease, RNA metabolism and cell adhesion. The Alzheimer's disease pathway encompasses many cellular processes including receptor signalling, oxidative phosphorylation and Ca signalling, which all lead to apoptosis. Alzheimer's disease has been associated with the metabolic syndrome in humans^(^^14^^)^ and Wistar Kyoto rats fed a HFD show increases in the levels of biomarkers for Alzheimer's disease^(^^15^^)^. Apoptosis was one of the main pathways that we reported for the analysis with all genes that were differentially expressed in the placentae of dams on the HFD or the control diet. Decreases in this pathway could result in reduced levels of apoptosis and could thereby also lead to increased placental and fetal size. Indeed, increased levels of placental apoptosis have previously been correlated to reduced placental size in primate and rodent models of maternal undernutrition^(^^12^^,^^16^^,^^17^^)^.

The arachidonic acid metabolism pathway was also significantly altered in the placentae of rats fed the HFD. COX2 belongs to this pathway and its mRNA and protein expression was increased in placentae from HFD-fed rat dams. mRNA for *Ptgs2*, *Ptgs1* and the PG-metabolising enzyme *Hpgd* was increased in the placentae of female but not male offspring of HFD-fed mouse dams at E12.5^(^^7^^)^. In humans, increased placental mRNA expression of *PTGS2* has been reported in pregnancies complicated with pre-eclampsia in some^(^^18^^,^^19^^)^ but not all studies^(^^20^^,^^21^^)^. This increase in expression was localised to the syncytiotrophoblast cells^(^^18^^)^. In pre-eclampsia, the vascularisation of the placenta is dysregulated^(^^22^^,^^23^^)^. Fetal polymorphisms in *PTGS2* have been shown to cause placental malperfusion in 132 twin pregnancies^(^^24^^)^, again indicating a role for COX2 in placental blood flow. The mRNA expression of *Pla2g2a*, which encodes for phospholipase A2, the enzyme catalysing the reaction from phosphatidylcholine to arachidonic acid and lysophosphatidylcholine, was also increased, providing substrate for COX2. A high gene expression of phospholipase A2 was also reported in placentae from obese neonates and was correlated to accumulation of *n*-3 fatty acids^(^^25^^)^. In the present study, neonatal circulating *n*-3 fatty acids and neonatal body composition were not measured.

Expression for LIMK1 showed the largest difference in expression between the control diet and the HFD. LIMK1 encodes a serine/threonine protein kinase which contains two LIM domains. It is a modulator of actin and microtubule dynamics^(^^26^^)^ and has been implicated in the mitotic process^(^^27^^)^. In the mouse, LIMK1 has been shown to be expressed early in the embryo as well as in trophoblast giant cells^(^^28^^)^.

A limitation of the present study is the relatively small sample size. This precluded an analysis of the effects of sex in the response to HFD feeding. In mice, genome-wide placental gene expression has been carried out and analysis has shown that HFD feeding resulted in differential gene expression of many genes in a sexually dimorphic pattern, with females showing larger changes than males^(^^6^^–^^8^^)^. In these studies, a slightly larger number of placentae or pools of placentae were studied for each condition and results were not corrected for multiple testing either. However, the present study found changes in similar genes^(^^7^^)^ and similar pathways^(^^8^^)^ to the studies in mice, indicating common effects of a HFD on placental gene expression.

## *Conclusion*

Maternal HFD feeding alters placental gene expression at E17.25, with pathways promoting inflammation and growth being over-represented among up-regulated genes and those affecting apoptosis among down-regulated genes. COX2, phospholipase A2 and LIMK1 mRNA and protein expression were up-regulated after maternal HFD feeding, implicating arachidonic acid and PG metabolism as targets of maternal diet effects.

## Supplementary Material

Supplementary Material Supplementary information supplied by authors.

## Supplementary material

To view supplementary material for this article, please visit http://dx.doi.org/10.1017/jns.2013.36
