# Supplementary material for: Maternal high-fat diet alters expression of pathways of growth, blood supply and arachidonic acid in rat placenta
Source: J Nutr Sci. 2014 Jan 2;2:e41. doi: 10.1017/jns.2013.36 (PMC4153320; doi:10.1017/jns.2013.36)
Supplement: Supplementary Material — Supplementary information supplied by authors. [file S2048679013000360sup001.pdf]

Supplementary table 1 Primer sequences for quantitative PCR

| Gene symbol | Forward primer (5'-3') | Reverse primer (5'-3') |
|---|---|---|
| *Ptgs2* | TCA CCC GAG GAC TGG GCC AT | CAG CGA ACC GCA GGT GCT CA |
| *Pla2g2a* | GCC ACA GAT TGG TGC TGT GTG A | TGG CCC CCT CGG TAG GAG AAC |
| *Serpine1* | CCT ACG GCG TGT CCT CGG TG | GAG CTT TCG GAG GGC AGC |
| *Umps* | AGC AGC AGT TGG GAT GGC TGA | TGC GAG TAT TCC CCG GCC CA |
| *Itga1* | AGC GAG CCC ACG GAA AAG CA | GCT CCC ACC GCT CCA AGC AT |
| *Limk1* | GGC CCT GAA TGC TGA CTG GCA | GTT CGG AGC ACC CGT GGC AA |
| *Actb* | TCC ACC CGC GAG TAC AAC CT | TTG CAC ATG CCG GAG CCG TT |

Supplementary Table 2. List of upregulated and downregulated genes in placentae of rat dams on high fat diet (HFD) *vs.* control diet (CD).

| **Upregulated genes**<br>**Gene Symbol** | **P value** | **Fold change**<br>HFD v CD | **Downregulated genes**<br>**Gene Symbol** | **P value** | **Fold change**<br>HFD v CD |
|---|---|---|---|---|---|
| *Limk1* | 0.0175 | 2.03 | *RGD1359529* | 0.0409 | 0.58 |
| *Raet1l* | 0.0023 | 1.92 | *Umps* | 0.0048 | 0.59 |
| *Chmp4bl1* | 0.0410 | 1.91 | *Vim* | 0.0283 | 0.67 |
| *Serpine1* | 0.0449 | 1.80 | *Slc4a4* | 0.0449 | 0.68 |
| *Cyp26b1* | 0.0190 | 1.59 | *RGD1309085* | 0.0188 | 0.69 |
| *Chdh* | 0.0413 | 1.57 | *RGD1306962* | 0.0030 | 0.69 |
| *Pf4* | 0.0227 | 1.55 | *App* | 0.0175 | 0.72 |
| *Bat2* | 0.0072 | 1.54 | *Phyh* | 0.0019 | 0.72 |
| *LOC499779* | 0.0201 | 1.51 | *Tcf21* | 0.0364 | 0.73 |
| *Itga1* | 0.0423 | 1.51 | *Bat5* | 0.0266 | 0.73 |
| *Bcl2a1d* | 0.0091 | 1.50 | *Rbm39* | 0.0160 | 0.74 |
| *Map2k6* | 0.0353 | 1.48 | *Ctnnb1* | 0.0058 | 0.74 |
| *Upp1* | 0.0490 | 1.48 | *Chkb* | 0.0208 | 0.74 |
| *Miox* | 0.0013 | 1.46 | *Gucy1b3* | 0.0444 | 0.74 |
| *Ptgs2* | 0.0438 | 1.44 | *Tardbp* | 0.0085 | 0.75 |
| *Igfbp2* | 0.0025 | 1.43 | *Lamb2* | 0.0494 | 0.75 |
| *Arl6ip5* | 0.0012 | 1.41 | *Fmr1* | 0.0210 | 0.76 |
| *St6galnac3* | 0.0427 | 1.40 | *RGD1565775* | 0.0371 | 0.76 |
| *Pla2g2a* | 0.0440 | 1.40 | *Elmo3* | 0.0243 | 0.78 |
| *Actn1* | 0.0058 | 1.39 | *LOC498750* | 0.0107 | 0.78 |
| *Zfp354b* | 0.0136 | 1.38 | *Zfp347* | 0.0086 | 0.79 |
| *Mmp9* | 0.0303 | 1.37 | *Gls2* | 0.0336 | 0.79 |
| *B4galt4* | 0.0472 | 1.36 | *Xpnpep1* | 0.0252 | 0.80 |
| *Rab3d* | 0.0362 | 1.36 | *LOC685878* | 0.0410 | 0.80 |
| *Degs1* | 0.0387 | 1.36 | *Taf1d* | 0.0264 | 0.80 |
| *Pctp* | 0.0319 | 1.35 | *Thra* | 0.0176 | 0.80 |
| *Qsox1* | 0.0303 | 1.35 | *RGD1561108* | 0.0494 | 0.80 |
| *Kcnk12* | 0.0408 | 1.34 | *Dhx15* | 0.0195 | 0.81 |
| *Slc27a1* | 0.0051 | 1.34 | *Smchd1* | 0.0154 | 0.81 |
| *Flnc* | 0.0462 | 1.34 | *Wnk1* | 0.0435 | 0.81 |
| *Hs3st2* | 0.0342 | 1.33 | *Dvl1* | 0.0071 | 0.82 |
| *Cd63* | 0.0089 | 1.33 | *Hs3st1* | 0.0391 | 0.82 |
| *Cd59* | 0.0239 | 1.33 | *Giot1* | 0.0171 | 0.82 |
| *Ero1l* | 0.0326 | 1.32 | *Fbxo11* | 0.0474 | 0.82 |
| *LOC689985* | 0.0129 | 1.32 | *Tmem37* | 0.0376 | 0.82 |
| *Slc6a2* | 0.0404 | 1.30 | *Iws1* | 0.0252 | 0.82 |
| *Rnf128* | 0.0256 | 1.30 | *Arfip2* | 0.0258 | 0.82 |
| *Aprt* | 0.0153 | 1.29 | *Ssbp1* | 0.0306 | 0.82 |
| *Ptgfrn* | 0.0466 | 1.29 | *Nt5dc2* | 0.0409 | 0.82 |
| *LOC307347* | 0.0042 | 1.29 | *Fip1l1* | 0.0391 | 0.82 |
| *Fmr1nb* | 0.0477 | 1.28 | *Cat* | 0.0319 | 0.83 |
| *Dnajb6* | 0.0079 | 1.27 | *RGD1563786* | 0.0248 | 0.83 |
| *LOC362526* | 0.0194 | 1.27 | *Ide* | 0.0280 | 0.83 |
| *Krt19* | 0.0137 | 1.27 | *Hsd17b8* | 0.0373 | 0.83 |
| *B3gnt7* | 0.0195 | 1.26 | *Sfrs2* | 0.0458 | 0.83 |
| *Yif1* | 0.0111 | 1.25 | *Znf667* | 0.0414 | 0.83 |
| *Spryd3* | 0.0023 | 1.25 | *Amacr* | 0.0132 | 0.84 |

| | | | | | |
|---|---|---|---|---|---|
| *Cgrrf1* | 0.0074 | 1.25 | *Tbce* | 0.0170 | 0.84 |
| *Ifi35* | 0.0498 | 1.25 | *RGD1560523* | 0.0164 | 0.84 |
| *Cln8* | 0.0402 | 1.25 | *Atp5b* | 0.0462 | 0.84 |
| *Spns1* | 0.0179 | 1.24 | *Gdi1* | 0.0478 | 0.84 |
| *Tap1* | 0.0111 | 1.24 | *Sarnp* | 0.0137 | 0.85 |
| *Tmed5* | 0.0018 | 1.24 | *Mllt4* | 0.0204 | 0.85 |
| *Sec61a1* | 0.0169 | 1.24 | *Ppp2r2d* | 0.0357 | 0.85 |
| *Sh2b2* | 0.0302 | 1.23 | *Hspa14* | 0.0259 | 0.85 |
| *Ifi27* | 0.0212 | 1.23 | *Aplf* | 0.0212 | 0.86 |
| *Cspg5* | 0.0006 | 1.23 | *Ythdc1* | 0.0327 | 0.86 |
| *Pla2g15* | 0.0133 | 1.23 | *Ptbp2* | 0.0392 | 0.86 |
| *Tap2* | 0.0439 | 1.23 | *Psma6* | 0.0446 | 0.86 |
| *Csrp1* | 0.0418 | 1.23 | *Tmem30a* | 0.0222 | 0.86 |
| *Lrrc8a* | 0.0264 | 1.23 | *Ostf1* | 0.0440 | 0.86 |
| *Tmed9* | 0.0490 | 1.22 | *RGD1564940* | 0.0099 | 0.86 |
| *Mthfs* | 0.0102 | 1.22 | *Snx16* | 0.0206 | 0.86 |
| *Cflar* | 0.0026 | 1.22 | *Exosc9* | 0.0323 | 0.87 |
| *Myadm* | 0.0375 | 1.22 | *Jup* | 0.0453 | 0.87 |
| *LOC688637* | 0.0039 | 1.22 | *Prl8a2* | 0.0253 | 0.87 |
| *Cd82* | 0.0055 | 1.22 | *Geft* | 0.0480 | 0.87 |
| *RGD1562758* | 0.0024 | 1.22 | *Puf60* | 0.0058 | 0.87 |
| *Ppt2* | 0.0376 | 1.21 | *Epc2l1* | 0.0244 | 0.87 |
| *Ppp3ca* | 0.0370 | 1.21 | *Thrb* | 0.0094 | 0.87 |
| *Cspg5* | 0.0210 | 1.21 | *Rgl2* | 0.0194 | 0.87 |
| *Rilpl2* | 0.0335 | 1.21 | *Nfyc* | 0.0270 | 0.88 |
| *Tmem192* | 0.0167 | 1.21 | *Cnbp* | 0.0251 | 0.88 |
| *Gemin8* | 0.0043 | 1.21 | *RGD1310592* | 0.0331 | 0.88 |
| *Dhrs9* | 0.0058 | 1.21 | *Slc25a3* | 0.0476 | 0.88 |
| *Vti1a* | 0.0356 | 1.21 | *Fbxo30* | 0.0493 | 0.89 |
| *RGD1303003* | 0.0033 | 1.21 | *Bat3* | 0.0473 | 0.89 |
| *Ctss* | 0.0403 | 1.21 | *Pcyt2* | 0.0258 | 0.89 |
| *Gna14* | 0.0475 | 1.20 | *Ccnyl1* | 0.0313 | 0.89 |
| *RGD1561113* | 0.0429 | 1.20 | *Rpp38* | 0.0086 | 0.89 |
| *Star* | 0.0200 | 1.20 | *Parn* | 0.0202 | 0.89 |
| *Tor1aip1* | 0.0007 | 1.20 | *Casp12* | 0.0233 | 0.89 |
| *Snd1* | 0.0252 | 1.20 | *Smc1a* | 0.0219 | 0.89 |
| *Thrap3* | 0.0322 | 1.19 | *Dtx3* | 0.0423 | 0.89 |
| *Lrrn2* | 0.0284 | 1.19 | *Rplp0* | 0.0236 | 0.90 |
| *Hint3* | 0.0142 | 1.19 | *Sfrs12* | 0.0376 | 0.90 |
| *rnf141* | 0.0006 | 1.19 | *Zhx1* | 0.0157 | 0.90 |
| *Dio3* | 0.0395 | 1.19 | *Pan2* | 0.0483 | 0.90 |
| *Rab11b* | 0.0142 | 1.19 | *RGD1562646* | 0.0442 | 0.91 |
| *Abca5* | 0.0223 | 1.19 | *RGD1304868* | 0.0434 | 0.91 |
| *Tmem43* | 0.0391 | 1.19 | *Zfp277* | 0.0306 | 0.91 |
| *Esyt1* | 0.0214 | 1.18 | *Cox7b* | 0.0160 | 0.91 |
| *G6pc3* | 0.0269 | 1.18 | *Mrps7* | 0.0462 | 0.91 |
| *Cyb561d2* | 0.0123 | 1.18 | *RGD1565131* | 0.0067 | 0.91 |
| *Gorasp2* | 0.0171 | 1.18 | *Vps16* | 0.0131 | 0.91 |
| *Trappc3* | 0.0104 | 1.18 | *Yme1l1* | 0.0379 | 0.91 |
| *RGD1562125* | 0.0183 | 1.18 | *Rpl32* | 0.0280 | 0.91 |
| *Nr1h2* | 0.0007 | 1.18 | *Ttc21b* | 0.0076 | 0.92 |
| *RGD1308626* | 0.0181 | 1.18 | *Abhd14a* | 0.0124 | 0.92 |
| *Hnrnpul2* | 0.0177 | 1.17 | *Fam126a* | 0.0175 | 0.92 |

| | | |
|---|---|---|
| *Tsku* | 0.0299 | 1.17 |
| *Nsmce1* | 0.0070 | 1.17 |
| *Znf688* | 0.0302 | 1.17 |
| *Timm22* | 0.0052 | 1.17 |
| *Fam96a* | 0.0106 | 1.17 |
| *Eif5a* | 0.0252 | 1.17 |
| *Unc13d* | 0.0387 | 1.17 |
| *Mcf2* | 0.0133 | 1.17 |
| *Tax1bp3* | 0.0091 | 1.17 |
| *Arf5* | 0.0091 | 1.17 |
| *Stard3* | 0.0106 | 1.16 |
| *Oaf* | 0.0079 | 1.16 |
| *Leprot* | 0.0032 | 1.16 |
| *Arf1* | 0.0343 | 1.16 |
| *Hnrnpa1* | 0.0286 | 1.16 |
| *Zdhhc14* | 0.0215 | 1.16 |
| *Pycrl* | 0.0087 | 1.16 |
| *Atrnl1* | 0.0200 | 1.16 |
| *Tyro3* | 0.0366 | 1.16 |
| *Slc38a10* | 0.0069 | 1.16 |
| *RGD1562987* | 0.0069 | 1.16 |
| *Emd* | 0.0205 | 1.16 |
| *Psmc3* | 0.0113 | 1.16 |
| *Dnajc18* | 0.0126 | 1.16 |
| *Vkorc1l1* | 0.0224 | 1.16 |
| *Nudt9* | 0.0206 | 1.16 |
| *Rc3h1* | 0.0355 | 1.16 |
| *Kcnn3* | 0.0005 | 1.16 |
| *Gapdh* | 0.0392 | 1.16 |
| *Pom121* | 0.0458 | 1.16 |
| *Cwc15* | 0.0161 | 1.16 |
| *Cript* | 0.0057 | 1.16 |
| *Faim* | 0.0390 | 1.15 |
| *Pigv* | 0.0399 | 1.15 |
| *RGD1305007* | 0.0325 | 1.15 |
| *Ndnl2* | 0.0104 | 1.15 |
| *Shisa5* | 0.0146 | 1.15 |
| *Ubl7* | 0.0372 | 1.15 |
| *Snip1* | 0.0192 | 1.15 |
| *Dopey2* | 0.0458 | 1.15 |
| *Tmem87a* | 0.0377 | 1.15 |
| *Enoph1* | 0.0409 | 1.15 |
| *Usp27x* | 0.0257 | 1.15 |
| *Etf1* | 0.0103 | 1.14 |
| *Nfkbil1* | 0.0106 | 1.14 |
| *Styxl1* | 0.0260 | 1.14 |
| *Wbp2* | 0.0015 | 1.14 |
| *Psmc5* | 0.0279 | 1.14 |
| *RGD1305235* | 0.0282 | 1.14 |
| *Arf6* | 0.0092 | 1.14 |
| *Tbc1d10b* | 0.0153 | 1.14 |
| *Diablo* | 0.0079 | 1.14 |
| *Ifit1lb* | 0.0483 | 1.14 |
| *Anks3* | 0.0391 | 0.92 |
| *Orc4l* | 0.0356 | 0.92 |
| *Batf3* | 0.0437 | 0.92 |
| *Mks1* | 0.0048 | 0.92 |
| *Ctsl1* | 0.0438 | 0.92 |
| *Grinl1a* | 0.0351 | 0.92 |
| *Lrrc14* | 0.0231 | 0.92 |
| *Zbtb4* | 0.0462 | 0.93 |
| *Pir* | 0.0301 | 0.93 |
| *Zfp418* | 0.0260 | 0.93 |
| *Otud4* | 0.0097 | 0.93 |
| *RGD1562874* | 0.0012 | 0.93 |
| *RGD1307986* | 0.0225 | 0.94 |
| *Mettl8* | 0.0293 | 0.94 |
| *Klhl18* | 0.0400 | 0.94 |
| *Cercam* | 0.0114 | 0.94 |
| *Sdr42e1* | 0.0311 | 0.94 |
| *Slc25a14* | 0.0447 | 0.94 |
| *Ankrd16* | 0.0203 | 0.95 |
| *RGD1561551* | 0.0299 | 0.95 |
| *Ppp1r14c* | 0.0471 | 0.95 |
| *Hoxc6* | 0.0464 | 0.95 |
| *Vav3* | 0.0314 | 0.95 |
| *Kel* | 0.0408 | 0.95 |
| *Fignl1* | 0.0487 | 0.96 |
| *RGD1562012* | 0.0212 | 0.96 |
| *Morn4* | 0.0411 | 0.96 |
| *RGD1566314* | 0.0347 | 0.97 |
| *Fam13c1* | 0.0433 | 0.97 |
| *Dpp4* | 0.0459 | 0.97 |

| | | |
|---|---|---|
| *LOC367191* | 0.0292 | 1.14 |
| *Nudc* | 0.0384 | 1.14 |
| *Dusp9* | 0.0128 | 1.14 |
| *Ccdc23* | 0.0298 | 1.14 |
| *Pim1* | 0.0082 | 1.14 |
| *Slc10a3* | 0.0396 | 1.14 |
| *Yipf1* | 0.0177 | 1.13 |
| *Pcbp2* | 0.0151 | 1.13 |
| *Bckdha* | 0.0195 | 1.13 |
| *Tanc2* | 0.0498 | 1.13 |
| *Ppp4c* | 0.0054 | 1.13 |
| *Zyg11b* | 0.0328 | 1.13 |
| *Coq4* | 0.0212 | 1.13 |
| *Tm2d2* | 0.0163 | 1.13 |
| *Fcrlb* | 0.0187 | 1.13 |
| *Pabpc1l2b* | 0.0146 | 1.13 |
| *LOC497934* | 0.0316 | 1.13 |
| *Tnpo1* | 0.0188 | 1.13 |
| *Cox17* | 0.0472 | 1.13 |
| *Thop1* | 0.0365 | 1.13 |
| *Prdx1* | 0.0260 | 1.13 |
| *Hnrnpk* | 0.0438 | 1.13 |
| *Stxbp5* | 0.0208 | 1.13 |
| *Tmed2* | 0.0195 | 1.13 |
| *Tssc1* | 0.0064 | 1.13 |
| *Axin1* | 0.0035 | 1.13 |
| *Zxdc* | 0.0188 | 1.12 |
| *Scamp2* | 0.0440 | 1.12 |
| *Rpl10a* | 0.0458 | 1.12 |
| *Commd10* | 0.0285 | 1.12 |
| *Ppp1r7* | 0.0008 | 1.12 |
| *Pias2* | 0.0017 | 1.12 |
| *Uxs1* | 0.0062 | 1.12 |
| *Cdc16* | 0.0119 | 1.12 |
| *Fam129a* | 0.0353 | 1.12 |
| *Pelp1* | 0.0242 | 1.12 |
| *Ndufa13* | 0.0477 | 1.12 |
| *Fam19a5* | 0.0264 | 1.12 |
| *Fam58b* | 0.0119 | 1.12 |
| *RGD1564364* | 0.0185 | 1.12 |
| *Arfrp1* | 0.0286 | 1.12 |
| *Cct4* | 0.0108 | 1.12 |
| *Gramd2* | 0.0120 | 1.12 |
| *Add1* | 0.0306 | 1.12 |
| *Soat1* | 0.0387 | 1.11 |
| *Fbn2* | 0.0393 | 1.11 |
| *Mblac1* | 0.0354 | 1.11 |
| *Tmem60* | 0.0417 | 1.11 |
| *Arfgef1* | 0.0484 | 1.11 |
| *Atp5h* | 0.0482 | 1.11 |
| *Exosc6* | 0.0339 | 1.11 |
| *Phox2a* | 0.0015 | 1.11 |
| *Nfya* | 0.0406 | 1.11 |

| | | |
|---|---|---|
| *Alg6* | 0.0088 | 1.11 |
| *Cdx1* | 0.0383 | 1.11 |
| *Cmpk1* | 0.0103 | 1.11 |
| *Olr1201* | 0.0070 | 1.11 |
| *Rasd2* | 0.0469 | 1.11 |
| *Banf1* | 0.0321 | 1.10 |
| *Eral1* | 0.0329 | 1.10 |
| *Slc35a4* | 0.0103 | 1.10 |
| *Cdkn2aipnl* | 0.0470 | 1.10 |
| *Rpusd4* | 0.0355 | 1.10 |
| *Rps3* | 0.0277 | 1.10 |
| *Ap3m2* | 0.0264 | 1.10 |
| *Zdhhc7* | 0.0205 | 1.10 |
| *Gcap14* | 0.0271 | 1.10 |
| *Itfg1* | 0.0298 | 1.10 |
| *Nrgn* | 0.0094 | 1.10 |
| *Rap1a* | 0.0037 | 1.10 |
| *Cct2* | 0.0484 | 1.10 |
| *Gip* | 0.0244 | 1.10 |
| *RGD1562953* | 0.0375 | 1.10 |
| *Dcbld2* | 0.0444 | 1.09 |
| *Gnb2* | 0.0422 | 1.09 |
| *Zfp143* | 0.0406 | 1.09 |
| *Fut2* | 0.0485 | 1.09 |
| *Epn2* | 0.0318 | 1.09 |
| *Tecpr1* | 0.0226 | 1.09 |
| *RGD1565368* | 0.0441 | 1.09 |
| *Rbm15b* | 0.0213 | 1.09 |
| *Atic* | 0.0182 | 1.09 |
| *Ccl17* | 0.0009 | 1.09 |
| *Shmt2* | 0.0224 | 1.09 |
| *Il8rb* | 0.0239 | 1.09 |
| *Nucks1* | 0.0430 | 1.09 |
| *Csprs* | 0.0352 | 1.09 |
| *Dffb* | 0.0077 | 1.09 |
| *Nanp* | 0.0177 | 1.09 |
| *Blmh* | 0.0409 | 1.09 |
| *Cabp7* | 0.0039 | 1.09 |
| *Zcchc10* | 0.0445 | 1.09 |
| *Paqr3* | 0.0177 | 1.09 |
| *Cyp4a10* | 0.0272 | 1.09 |
| *Tfpt* | 0.0018 | 1.09 |
| *Tsc1* | 0.0056 | 1.09 |
| *Dbn1* | 0.0351 | 1.09 |
| *B4galnt1* | 0.0364 | 1.09 |
| *Scyl2* | 0.0347 | 1.09 |
| *N4bp2l2* | 0.0244 | 1.08 |
| *Tlr2* | 0.0294 | 1.08 |
| *Lrrc15* | 0.0040 | 1.08 |
| *Dhrs7b* | 0.0430 | 1.08 |
| *H1f0* | 0.0313 | 1.08 |
| *Cdc42bpb* | 0.0421 | 1.08 |
| *Srgap3* | 0.0385 | 1.08 |

| | | |
|---|---|---|
| *Mcpt8l3* | 0.0070 | 1.08 |
| *LOC501386* | 0.0082 | 1.08 |
| *Tlx3* | 0.0066 | 1.08 |
| *Rpl6* | 0.0460 | 1.08 |
| *Rassf8* | 0.0495 | 1.08 |
| *MGC94207* | 0.0236 | 1.08 |
| *RGD1562449* | 0.0224 | 1.08 |
| *Elac2* | 0.0385 | 1.08 |
| *Arntl* | 0.0200 | 1.07 |
| *Drd2* | 0.0061 | 1.07 |
| *Tgfbr1* | 0.0031 | 1.07 |
| *Tmem102* | 0.0438 | 1.07 |
| *Timm9* | 0.0065 | 1.07 |
| *RGD1565456* | 0.0293 | 1.07 |
| *Cyp2u1* | 0.0390 | 1.07 |
| *Fndc5* | 0.0008 | 1.07 |
| *Dyx1c1* | 0.0500 | 1.07 |
| *Mcpt1l2* | 0.0484 | 1.07 |
| *Pomc* | 0.0499 | 1.07 |
| *Mapk8* | 0.0140 | 1.07 |
| *LOC498470* | 0.0355 | 1.07 |
| *Hmgb3* | 0.0059 | 1.07 |
| *Zcwpw1* | 0.0062 | 1.07 |
| *Slc25a27* | 0.0467 | 1.06 |
| *Mllt11* | 0.0434 | 1.06 |
| *Cyhr1* | 0.0362 | 1.06 |
| *Tmtc1* | 0.0354 | 1.06 |
| *Slc2a13* | 0.0281 | 1.06 |
| *Hs1bp3* | 0.0035 | 1.06 |
| *RGD1559767* | 0.0410 | 1.06 |
| *Rbm43* | 0.0333 | 1.06 |
| *Atxn7* | 0.0015 | 1.06 |
| *Fhl4* | 0.0115 | 1.06 |
| *Cyp8b1* | 0.0027 | 1.06 |
| *LOC686141* | 0.0131 | 1.06 |
| *Zfp407* | 0.0443 | 1.06 |
| *Cacna1c* | 0.0308 | 1.06 |
| *Lrrc30* | 0.0048 | 1.06 |
| *Baalc* | 0.0313 | 1.06 |
| *RT1-Db1* | 0.0212 | 1.06 |
| *Slc44a1* | 0.0341 | 1.05 |
| *Nap5* | 0.0317 | 1.05 |
| *Nkx1-1* | 0.0127 | 1.05 |
| *RGD1560069* | 0.0435 | 1.05 |
| *Pcp4* | 0.0289 | 1.05 |
| *Fkrp* | 0.0183 | 1.05 |
| *LOC500705* | 0.0176 | 1.05 |
| *Pdyn* | 0.0402 | 1.05 |
| *Ntn5* | 0.0215 | 1.05 |
| *Capn1* | 0.0275 | 1.05 |
| *Ascl3* | 0.0030 | 1.04 |
| *Fam69b* | 0.0385 | 1.04 |
| *RGD1560050* | 0.0192 | 1.04 |

| | | |
|---|---|---|
| *Doxl2* | 0.0285 | 1.04 |
| *Btg4* | 0.0262 | 1.04 |
| *LOC688273* | 0.0319 | 1.04 |
| *Olr1437* | 0.0215 | 1.04 |
| *Slc2a1* | 0.0240 | 1.04 |
| *Serpina6* | 0.0173 | 1.04 |
| *Tshr* | 0.0271 | 1.04 |
| *Arrb1* | 0.0096 | 1.04 |
| *Olr157* | 0.0438 | 1.04 |
| *RGD1307694* | 0.0455 | 1.04 |
| *Nfasc* | 0.0423 | 1.04 |
| *Cplx1* | 0.0442 | 1.04 |
| *RGD1565482* | 0.0340 | 1.04 |
| *Olr484* | 0.0250 | 1.03 |
| *Ddn* | 0.0357 | 1.03 |

Supplementary table 3. List of upregulated genes with a fold change > 1.4 and downregulated genes with a fold change < 0.7 included in the pathway analysis

| **Upregulated genes Gene symbol** | **P value** | **Fold change** HFD *vs* CD | | **Downregulated genes Gene symbol** | **P value** | **Fold change** HFD *vs* CD |
|---|---|---|---|---|---|---|
| *Limk1* | 0.018 | 2.03 | | *App* | 0.018 | 0.72 |
| *Raet1l* | 0.023 | 1.92 | | *RGD1306962* | 0.003 | 0.69 |
| *Chmp4bl1* | 0.041 | 1.91 | | *RGD1309085* | 0.019 | 0.69 |
| *Serpine1* | 0.045 | 1.80 | | *Slc4a4* | 0.045 | 0.68 |
| *Cyp26b1* | 0.019 | 1.59 | | *Vim* | 0.028 | 0.67 |
| *Chdh* | 0.041 | 1.57 | | *Umps* | 0.005 | 0.60 |
| *Pf4* | 0.02 | 1.55 | | *RGD1359529* | 0.041 | 0.58 |
| *Bat2* | 0.007 | 1.54 | | | | |
| *LOC499779* | 0.02 | 1.51 | | | | |
| *Itga1* | 0.04 | 1.51 | | | | |
| *Bcl2a1d* | 0.009 | 1.50 | | | | |
| *Map2k6* | 0.035 | 1.48 | | | | |
| *Upp1* | 0.049 | 1.48 | | | | |
| *Miox* | 0.001 | 1.46 | | | | |
| *Ptgs2* | 0.043 | 1.44 | | | | |
| *Igfbp2* | 0.002 | 1.43 | | | | |
| *Arl6ip5* | 0.001 | 1.41 | | | | |
| *St6galnac3* | 0.043 | 1.40 | | | | |
| *Pla2g2a* | 0.044 | 1.40 | | | | |
